# Supplementary material for: Plastid genomics of Nicotiana (Solanaceae): insights into molecular evolution, positive selection and the origin of the maternal genome of Aztec tobacco (Nicotiana rustica)
Source: PeerJ. 2020 Jul 23;8:e9552. doi: 10.7717/peerj.9552 (PMC7382938; doi:10.7717/peerj.9552)
Supplement: Table S2 [file peerj-08-9552-s002.docx]

**Table S2 –** List of genes found in the plastid genomes of *Nicotiana knightiana, N. rustica, N. paniculata, N. obtusifolia* and *N. glauca.* Bolded genes were used in phylogenetic analysis.

| **Category**  **for gene** | **Group of gene** | **Name of gene** | | | | |
| --- | --- | --- | --- | --- | --- | --- |
| **Photosynthesis-related genes** | Photosystem Ⅰ | ***psaA*** | ***psaB*** | ***psaC*** | ***psaI*** | ***psaJ*** |
|  | Photosystem Ⅱ | ***psbA*** | ***PsbB*** | ***psbC*** | ***psbD*** | ***psbE*** |
|  |  | ***psbF*** | ***psbH*** | ***psbI*** | ***psbJ*** | ***psbK*** |
|  |  | ***psbL*** | ***psbM*** | ***psbN*** |  |  |
|  | Cytochrome  b/f compelx | ***psbT*** | ***psbZ*** | ***petN*** | ***petA*** | ***petL*** |
|  |  | ***petG*** | ***petD**** | ***petB**** |  |  |
|  | ATP synthase | ***atpI*** | ***atpH*** | ***atpA*** | ***atpF**** | ***atpE*** |
|  |  | ***atpB*** |  |  |  |  |
|  | Cytochrome  c synthesis | ***ccsA*** |  |  |  |  |
|  | Assembly/stability  of photosystem Ⅰ | ***ycf3******* | ***ycf4*** |  |  |  |
|  | NADPH dehydrogenase | ***ndhB*,^a,^*** | ***ndhH*** | ***ndhA**** | ***ndhI*** | ***ndhG*** |
|  |  | ***ndhJ*** | ***ndhE*** | ***ndhF*** | ***ndhC*** | ***ndhK*** |
|  |  | ***ndhD*** |  |  |  |  |
|  | Rubisco | ***rbcL*** |  |  |  |  |
| **Transcription**  **and translation**  **related genes**  **RNA genes** | Transcription  Small subunit  of ribosome | ***rpoA*** | ***rpoC2*** | ***rpoC1**** | ***rpoB*** | ***rps16**** |
|  |  | ***rps7^a,^*** | ***rps15*** | ***rps19*** | ***rps3*** | ***rps8*** |
|  |  | ***rps14*** | ***rps11*** | ***rps12^a,^**** | ***rps18*** | ***rps4*** |
|  |  | ***rps2*** |  |  |  |  |
|  | Large subunit  of ribosome | ***rpl2^a,^**** | ***rpl23^a,^*** | ***rpl32*** | ***rpl22*** | ***rpl14*** |
|  |  | ***rpl33*** | ***rpl36*** | ***rpl20*** | ***rpl16**** |  |
|  | Translational initiation factor | *infA* |  |  |  |  |
|  | Ribosomal RNA | *rrn16^a^,* | *rrn4.5^a^,* | *rrn5 ^a,^* | *rrn23^a^* |  |
|  | Transfer RNA | *trnV-GAC^a^* | *^.^ trnI-CAU^,^** | *trnA-UGC^a,^** | *trnN-GUU^a^* | *trnP-UGG* |
|  |  | *trnW-CCA,* | *trnV-UAC** | *trnL-UAA** | *trnF-GAA* | *trnRACG^a^* |
|  |  | *trnT-UGU* | *trnG-UCC^a,,^*^,^* | *trnT-GGU* | *trnR-UCU* | *trnE-UUC* |
|  |  | *trnY-GUA* | *trnD-GUC* | *trnC-GCA* | *trnS-GCU* | *trnH-GUG* |
|  |  | *trnK-UUU* | *trnQ-UUG* | *trnfM-CAU* | *trnG-GCC* | *trnS -UGA* |
|  |  | *trnS -GGA* | *trnF-GAA* | *trnM-CAU* | *trnL-CAA** |  |
|  |  | *trnI-GAU*^,a^* | *trnL -UAG* |  |  |  |
| **Other genes** | RNA processing | ***matK*** |  |  |  |  |
|  | Carbon metabolism | ***cemA*** |  |  |  |  |
|  | Fatty acid synthesis | *accD* |  |  |  |  |
|  | Proteolysis | ***clpP******* |  |  |  |  |
|  | Component of TIC complex | *ycf1^a^* |  |  |  |  |
|  | Hypothetical proteins | *ycf2^a^* |  |  |  |  |

* Gene with one intron, ** Gene with two introns, **^a^** Gene with two copies, Same genes in all *Nicotiana* species.
