# Supplementary figures and images for: Plastid genomics of Nicotiana (Solanaceae): insights into molecular evolution, positive selection and the origin of the maternal genome of Aztec tobacco (Nicotiana rustica)
Source: PeerJ. 2020 Jul 23;8:e9552. doi: 10.7717/peerj.9552 (PMC7382938; doi:10.7717/peerj.9552)

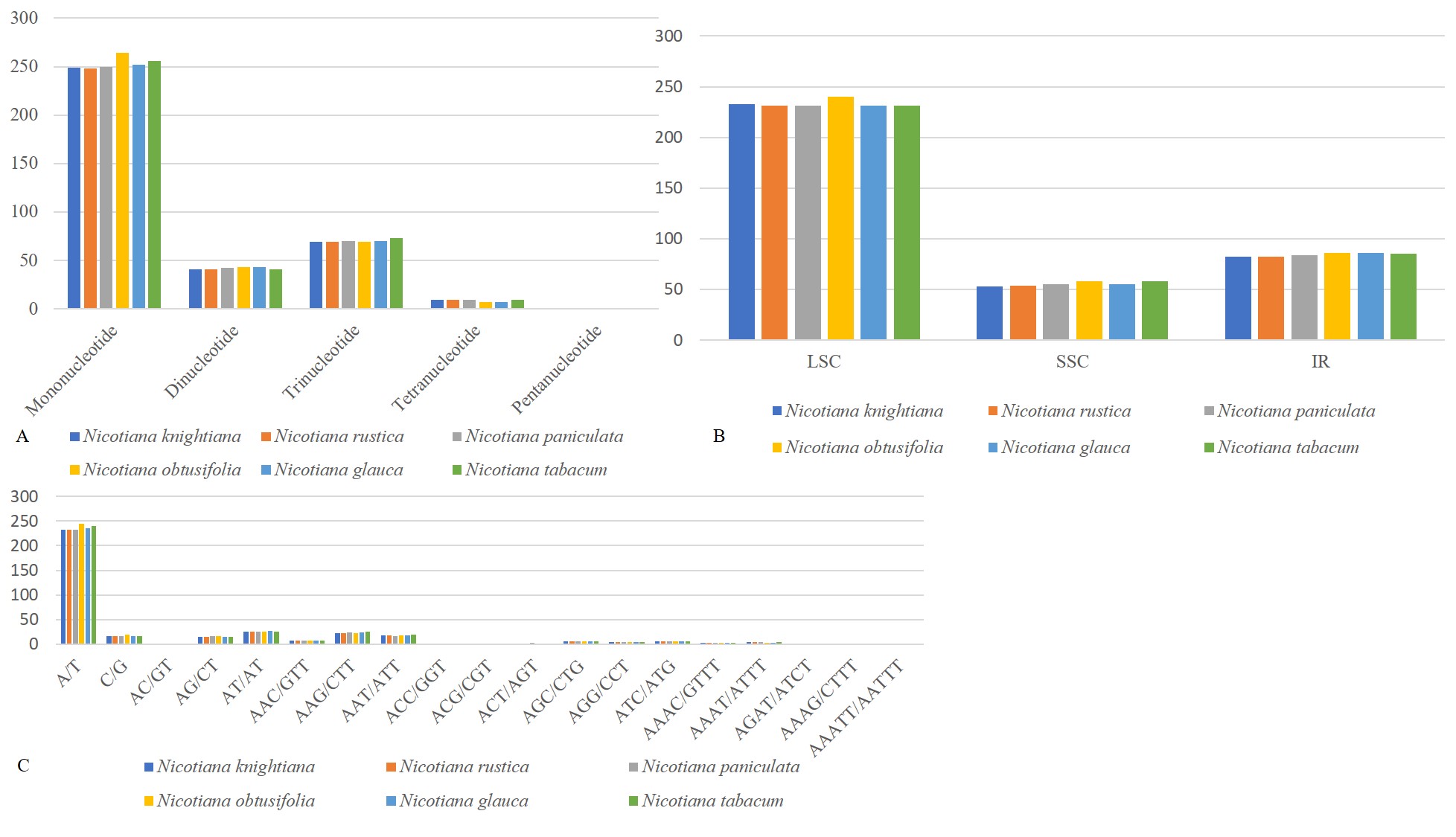

Supplement: Figure S1 — (A) Indicate numbers of various types of microsatellites present in the plastid genome of Nicotiana species. (B) Distribution of SSRs in different regions of the plastid genome of Nicotiana species. (C) SSRs motifs distribution in different regions of the plastid genome of Nicotiana species. [file peerj-08-9552-s010.jpg]

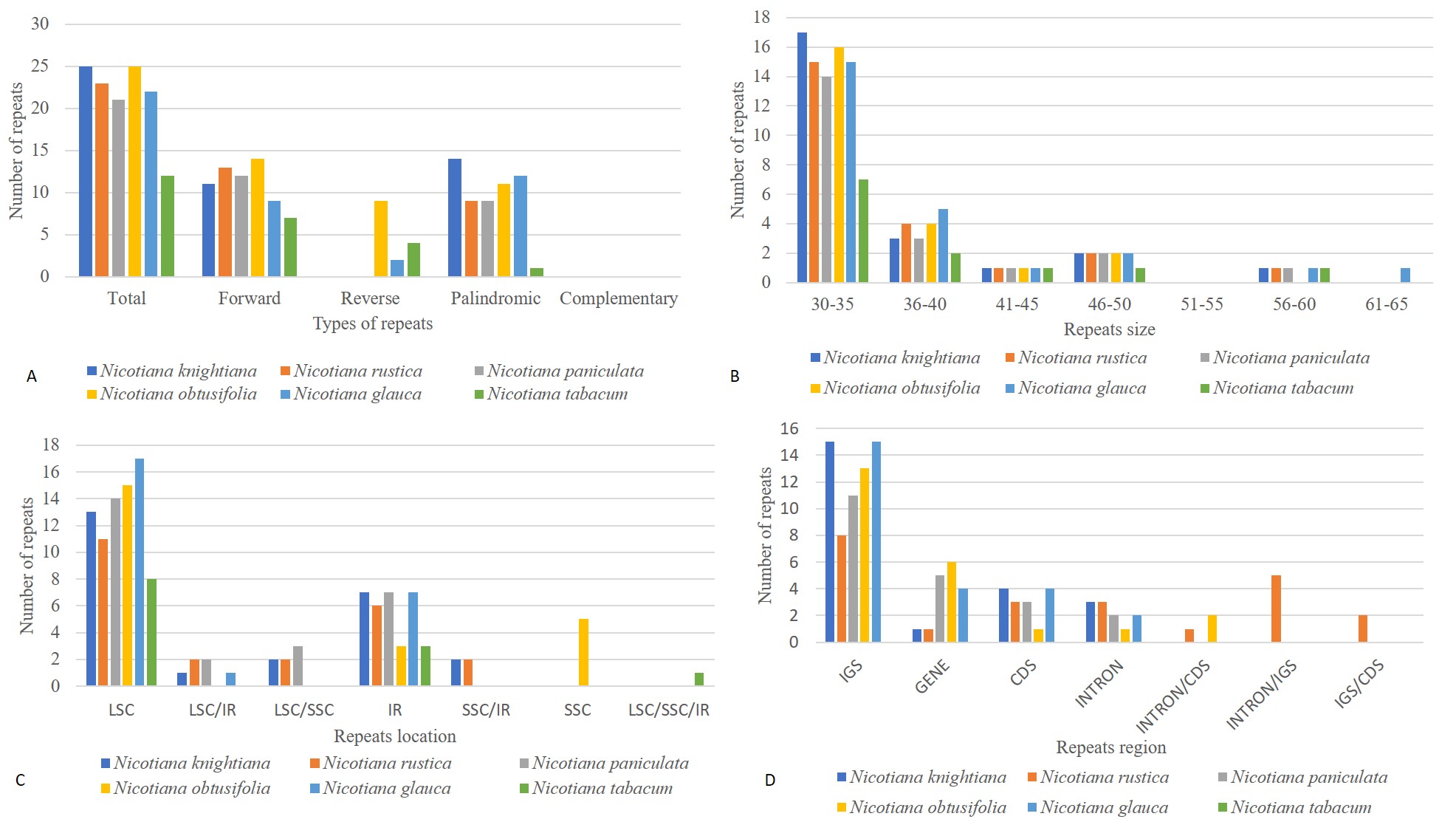

Supplement: Figure S2 [file peerj-08-9552-s011.jpg]

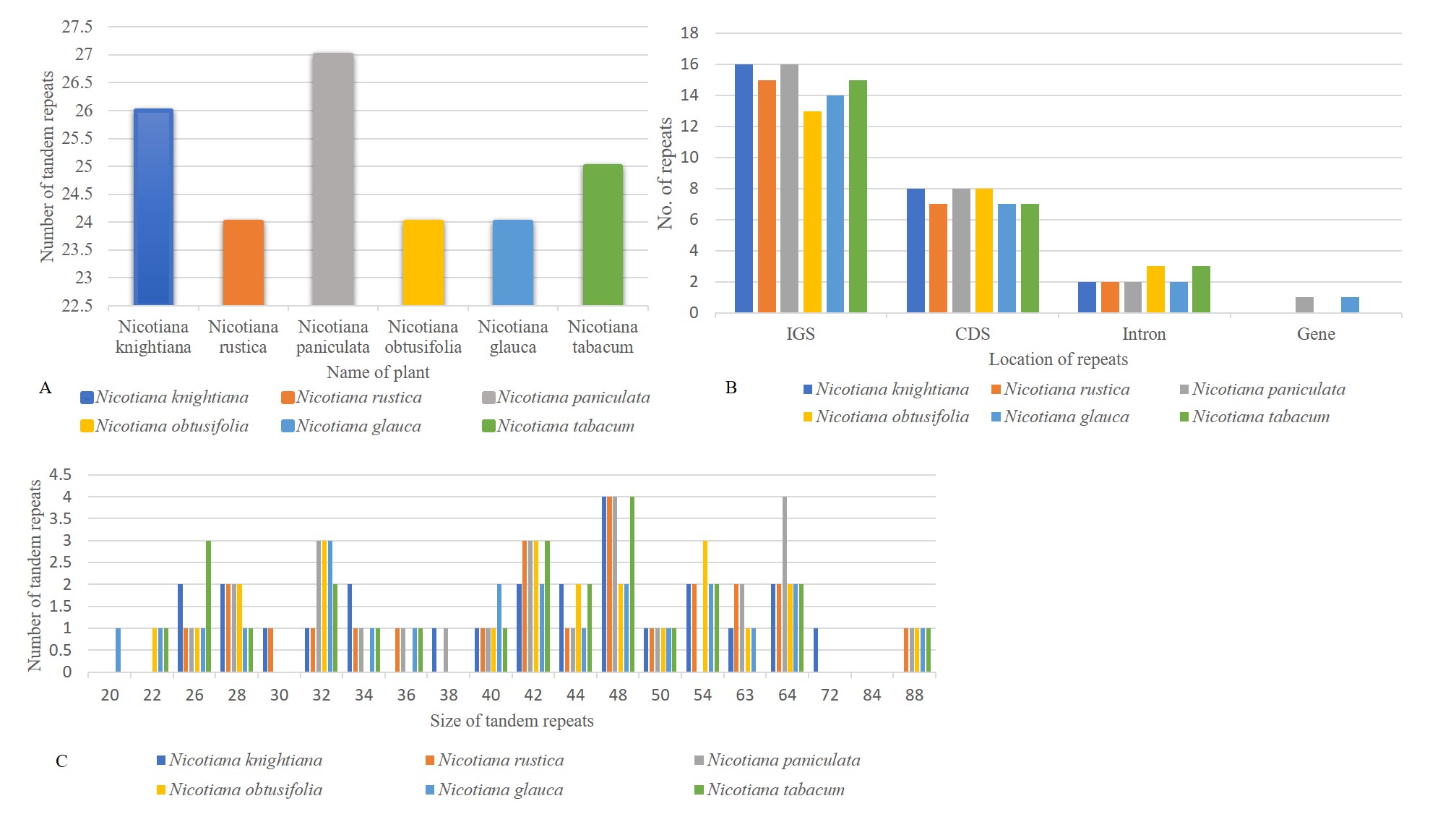

Supplement: Figure S3 [file peerj-08-9552-s012.jpg]
